# Unveiling the Rare: Sternoclavicular Joint Brucellosis Osteomyelitis—A Case Report and Literature Review

**DOI:** 10.1155/crdi/6674464

**Published:** 2025-11-04

**Authors:** Farhang Babamahmoodi, Rana Kolahi Ahari, Abdolreza Babamahmoodi, Tahereh Zareie

**Affiliations:** ^1^Antimicrobial Resistance Research Center, Communicable Diseases Institute, Mazandaran University of Medical Sciences, Sari, Iran; ^2^Innovative Medical Research Center, MMS.C., Islamic Azad University, Mashhad, Iran

**Keywords:** brucellosis, osteomyelitis, sternoclavicular joint

## Abstract

Brucellosis, an endemic zoonotic infection, often presents with musculoskeletal complications, but sternoclavicular joint (SCJ) osteomyelitis is exceptionally rare. We report a case of a 49-year-old male with SCJ osteomyelitis secondary to brucellosis, emphasizing the diagnostic challenges and management. Advanced imaging and serology were pivotal in confirming osteomyelitis, underscoring the need for clinical suspicion in endemic regions. The patient achieved full recovery with a combination of antibiotics. This case highlights the importance of differentiating osteomyelitis from arthritis in atypical presentations.

## 1. Introduction

Brucellosis remains a significant public health concern in endemic regions such as the Middle East. While osteoarticular involvement occurs in 10%–85% of cases, sternoclavicular joint (SCJ) osteomyelitis is rare (1%-2%) [1, 2].

In endemic regions, brucellosis is the most common zoonotic infection, with an estimated 500,000 new human cases reported annually [3]. In humans, brucellosis can affect any organ system, including the musculoskeletal, cardiac, hepatic, reproductive, and nervous systems, often mimicking a variety of infectious and noninfectious diseases [4]. Although musculoskeletal manifestations such as arthralgia, septic arthritis, spondylitis, and osteomyelitis are frequently reported [5], involvement of the SCJ is an uncommon complication [1]. This report details a rare case of SCJ osteomyelitis, reviews the relevant literature, and discusses diagnostic and therapeutic strategies.

## 2. Case Presentation

A 49-year-old male from a rural region of northern Iran presented to our facility with a 2-week history of a painful right SCJ mass and significantly restricted right shoulder mobility. This was preceded by a 1-month prodrome of intermittent fever, night sweats, anorexia with an associated 5 kg weight loss, and migratory arthralgia, initially affecting the left knee before progressing to the right knee and wrists. His history was significant for recent consumption of unpasteurized dairy products.

**Figure 1:**
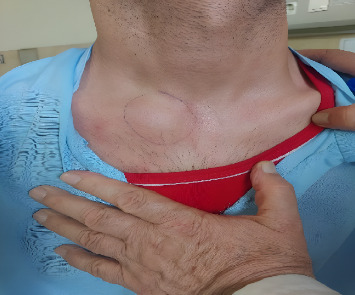
A warm, erythematous, and tender mass over the right sternoclavicular joint at admission.

Physical examination on admission revealed stable vital signs. A notable finding was a warm, tender, ill-defined 3.5 × 3 cm mass over the right SCJ, fixed to the underlying clavicle, with overlying erythema (Figure 1). The right shoulder active range of motion was reduced to approximately 50% of normal. Examination also revealed bilateral knee and wrist tenderness with mild effusion.

## 3. Diagnostic Workup

**Figure 2:**
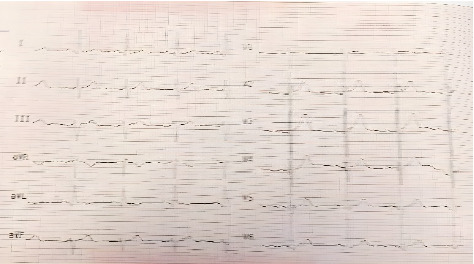
Preadmission ECG.

**Figure 3:**
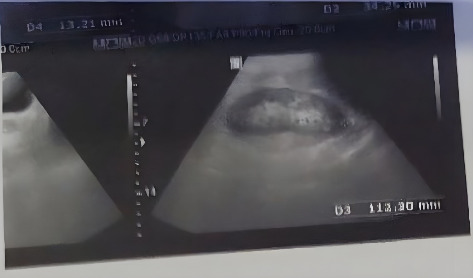
Abdominopelvic sonography showing a 13 mm echogenic mass (hemangioma) in the right lobe of the liver.

Initial laboratory investigations were significant for markedly elevated inflammatory markers (Table 1): C-reactive protein (CRP) 72 mg/L and erythrocyte sedimentation rate (ESR) 41 mm/hr. Serological testing confirmed brucellosis with a Wright agglutination titer of 1:640 and a 2-mercaptoethanol (2-ME) titer of 1:160. An electrocardiogram (ECG) was performed to rule out cardiac causes for the chest discomfort and revealed no abnormalities (Figure 2). A dedicated computed tomography (CT) scan of the SCJ demonstrated features consistent with osteomyelitis, including cortical erosion (8 mm depth), significant synovial hypertrophy (12 mm thickness), and a periarticular abscess (15 × 10 mm) (Figure 3). Blood cultures were negative. An abdominopelvic sonography, performed to investigate weight loss, incidentally identified a 13 mm hepatic lesion; subsequent MRI confirmed this to be a stable hemangioma with no contrast enhancement, and liver function tests remained normal. Other investigations, including TB QuantiFERON, interferon-gamma release assays, and tumor markers (CEA, CA19-9), were negative.

## 4. Treatment, Clinical Course, and Follow-Up

The patient was diagnosed with brucellosis with SCJ osteomyelitis and reactive polyarthritis. Treatment was initiated with a standard dual-therapy regimen of oral rifampicin (600 mg/day) and doxycycline (100 mg twice daily), supplemented with gentamicin (3 mg/kg/day) for the first week. Gentamicin was discontinued after 1 week due to a rise in serum creatinine (from a baseline of 0.8 to 1.4 mg/dL) and was substituted with oral ciprofloxacin to maintain effective bone penetration while mitigating nephrotoxicity. Adjunctive NSAIDs were used for anti-inflammatory and analgesic effects.

The clinical response was favorable and rapid. The migratory arthralgia and joint effusions resolved completely by the third week of therapy. The SCJ mass showed significant improvement, with a 50% reduction in size and tenderness by Day 10, culminating in complete resolution by Week 4. The patient completed a 6-week course of antimicrobial therapy. The patient's clinical presentation, diagnostic milestones, and response to treatment are summarized in Table 2.

At the 6-week follow-up, the patient was asymptomatic with near-complete restoration of shoulder mobility (90% of normal). Radiographic follow-up showed resolution of the osteomyelitic lesions, and inflammatory markers had normalized. He remained well at the 6-month follow-up with full functional capacity.

## 5. Discussion

### 5.1. Literature Review Methodology

We conducted a PubMed and Google Scholar search (2000–2024) using keywords “sternoclavicular brucellosis” and “osteomyelitis,” prioritizing cases with imaging-confirmed osteomyelitis. Of 32 identified studies, 12 met the inclusion criteria (Table 3).

### 5.2. Global Case Distribution

Most reported cases originate from brucellosis-endemic regions such as Iran, Turkey, and Peru. Notably, Berrocal et al. [6] reported 7 SCJ cases in Peru, while Turgut et al. highlighted spinal brucellosis in 6% of Turkish cases [7].

Brucellosis is transmitted to humans through close contact with infected animals, consumption of unpasteurized dairy products, or inhalation of airborne pathogens [8]. Although our patient denied direct animal contact, his history of traveling to rural areas and consuming unpasteurized dairy products is a recognized risk factor.

The infection typically presents with nonspecific symptoms such as fever, night sweats, myalgia, and arthralgia and can lead to localized infections in various organs [9]. Osteoarticular involvement is the most common complication, occurring in 10%–85% of cases, manifesting as sacroiliitis, peripheral arthritis, bursitis, or osteomyelitis [10]. Involvement is typically in weight-bearing joints such as the hip, knee, and vertebrae. SCJ involvement is rare, found in only 1%-2% of cases [2] and in 4.5% of cases in one Iranian study [11]. This rarity underscores the need for heightened clinical awareness.

The pathogenesis of *Brucella* osteomyelitis is not fully understood but is thought to involve hematogenous spread or local extension from mechanical stress, leading to inflammation, abscess formation, and impaired joint function [4, 11].

SCJ involvement has been infrequently reported. Recent cases include a middle-aged woman from Iran with a breast abscess and SCJ involvement [12], a 34 year-old Iranian female with osteochondritis diagnosed despite an initially negative Wright test [13], and a 31 year-old male from Iran with SCJ pain and redness [1]. A case from Turkey involved a woman with chest pain misdirected toward cardiac disease, who improved rapidly with brucellosis treatment [14]. Other reports include oligoarthritis involving the SCJ [15] and a case from Turkey requiring the addition of ciprofloxacin for symptom resolution [16].

Berrocal and his team analyzed cases over 21 years, finding seven instances of sternoclavicular arthritis among 1729 brucellosis patients. Most presented acutely, some with multijoint involvement, but all achieved full recovery with timely treatment, emphasizing the importance of early diagnosis [6].

A study of 452 brucellosis patients found osteoarticular complications in 169, with the hip (53%) and knee (36%) most commonly involved. The SCJ was affected in only 1.8% of cases [17].

Imaging is crucial for diagnosis. Denath [18] described CT findings for SCJ brucellosis, including a periarticular soft-tissue mass, bony sclerosis or lysis, and cortical destruction. These features, combined with clinical data, are key to diagnosing this severe form of brucellosis.

The diagnosis of *Brucella* osteomyelitis requires a multimodal approach, including serology, advanced imaging (MRI/CT), and sometimes biopsy for culture [19, 20]. Treatment involves prolonged combination antibiotic therapy [21]. Our patient responded favorably to a conventional regimen of doxycycline, rifampicin, and a brief course of gentamicin, which was switched to ciprofloxacin, resulting in a successful recovery.

Unlike some previously reported cases with multijoint involvement [15, 16], our case represents isolated SCJ osteomyelitis without concomitant arthritis, highlighting brucellosis' ability to manifest as a focal bone infection.

It is also important to note that brucellosis can have neurological manifestations (neurobrucellosis), which, although not present in our case, is a serious complication that clinicians must consider in the differential diagnosis [22, 23].

## 6. Conclusion

Although SCJ osteomyelitis remains uncommon, it demands heightened clinical suspicion in brucellosis-endemic regions. Collective evidence confirms that early diagnosis, achieved through advanced imaging and meticulous serology, enables prompt antibiotic therapy, yielding excellent response rates and favorable outcomes while preventing chronic complications.

### 6.1. Future Directions

Longitudinal studies are needed to optimize imaging protocols and antibiotic duration. Clinicians in endemic regions should consider brucellosis in the differential diagnosis for SCJ masses unresponsive to conventional anti-inflammatory therapy.

## Figures and Tables

**Table 1:** Pertinent laboratory findings on admission.

Parameter	Value	Normal range
White blood cell (WBC) count	8.4 × 10^3^/μL	4–10 × 10^3^/μL
C-Reactive protein (CRP)	72 mg/L	< 5 mg/L
Erythrocyte sedimentation rate (ESR)	41 mm/hr	< 15 mm/hr
Wright agglutination test	1:640	Negative < 1:80
2-Mercaptoethanol (2-ME) test	1:160	

**Table 2:** Timeline of clinical presentation, management, and resolution.

Time period	Clinical event	Diagnostic/treatment milestone
∼1 month preadmission	Consumption of unpasteurized dairy products	Antecedent risk factor
Month 1 (Prodromal)	Onset of fever, night sweats, migratory arthralgia (knees and wrists), weight loss (5 kg)	

Week 3 of illness	Development of a painful, swollen mass over the right sternoclavicular joint (SCJ)	

Week 4 (admission)	Presentation: SCJ mass, restricted shoulder motion (50% normal)	Diagnosis: elevated CRP/ESR, positive *Brucella* serology, CT-confirmed SCJ osteomyelitis
		Treatment start: rifampicin, doxycycline, gentamicin
Day 10 of treatment		50% reduction in size and tenderness of SCJ mass

Week 1 of treatment		Gentamicin discontinued due to rising creatinine; switched to ciprofloxacin

Week 3 of treatment	Complete resolution of bilateral knee/wrist tenderness and effusion	

Week 4 of treatment	Complete resolution of the SCJ mass	

Week 6 (end of therapy)	Asymptomatic; shoulder mobility improved to 90% of normal	Radiographic resolution of SCJ lesions; normalization of serology

6-Month follow-up	Remained asymptomatic; full shoulder mobility	Normal CRP

**Table 3:** Summary of case reports on sternoclavicular joint brucellosis.

Author and year	Country	Key findings	Serological results	Ref
Varshochi et al., 2024	Iran	SCJ arthritis and breast abscess	Wright: 1:160	[13]
Metanat et al., 2016	Iran	Osteochondritis of the SCJ	Wright: 1:640; 2-ME: 1:80	[14]
Shahabinejad et al. ,2015	Iran	Acute arthritis of the SCJ	Wright and Coombs: 1:640	[1]
Şaş et al., 2016	Turkey	Arthritis of the SCJ	Wright: 1:320	[16]
Alpay et al., 2017	Turkey	SCJ arthritis and biceps tenosynovitis	Wright: 1:160	[18]
Adak et al., 1997	Turkey	Oligoarthritis (TMJ, SCJ, ankle)	N/R	[17]
Berrocal et al., 1993	Peru	Case series (7 patients)	N/R	[6]
Mousa et al., 1987	Kuwait	Case series (169 OA cases)	N/R	[19]
Denath, 1991	South Africa	Radiological study (3 patients)	N/R	[20]
